# Severe dyspnea and uncontrolled seizures following meperfluthrin poisoning: a case report

**DOI:** 10.1186/s12887-021-02509-2

**Published:** 2021-01-25

**Authors:** Shengkun Zheng, Shengxin Zhang, Shaoxian Hong, Qing Lou

**Affiliations:** 1 Department of Emergency, Xiamen Children’s Hospital, 92-98 N Yibin road, Huli District, Xiamen, 361006 Fujian province China; 2 Department of Pediatric Intensive Care Unit, Xiamen Children’s Hospital, 92-98 N Yibin road, Huli District, Xiamen, 361006 Fujian province China

**Keywords:** Child, Meperfluthrin, Poisoning, Dyspnea, Seizures

## Abstract

**Background:**

Meperfluthrin is a novel sanitary cyhalothrin insecticide invented in China and has increasingly been used to produce liquid mosquito repellents. Oral meperfluthrin poisoning in human has rarely been reported. Here, we reported a case of meperfluthrin poisoning by ingestion of a meperfluthrin-based liquid mosquito repellent in a 16-month-old infant.

**Case presentation:**

A 16-month-old boy with a history of accident ingestion of meperfluthrin was admitted to our hospital’s emergency department. He exhibited severe dyspnea, and lung radiograph showed multiple patchy and cord-like high-density shadows bilaterally in a short time. He also suffered 35 min of seizures which were finally controlled by the intravenous infusion of propofol. He was diagnosed with meperfluthrin poisoning, status epilepticus and severe pneumonia. After treated with methylprednisolone, aerosolized beclomethasone dipropionate, anti-infection, and some critical supportive therapy, the patient was in good health and showed no symptoms during 12 months of follow-up.

**Conclusions:**

Meperfluthrin poisoning is rare. Oral meperfluthrin poisoning shows neurotoxic effects and pulmonary toxicity. Controlling seizures rapidly and ensuring an adequate oxygen supply are critical to the successful treatment.

## Background

Meperfluthrin is a novel sanitary cyhalothrin insecticide belonging to the pyrethroids and it has a high liquid solubility and hydrophobicity [1]. It is invented by Jiangsu Yangnong Chemical Company Limited (Co. Ltd) in China and belongs to a fluorine-containing insecticide with optical isomers [1]. Due to the nature of optically active isomer, meperfluthrin shows high insecticidal activities with less dosage, and its toxicity to non-target organisms has been further reduced [1].

Meperfluthrin has increasingly been used to produce liquid mosquito repellents because of its low toxicity, high efficiency [2, 3]. However, recent researches have revealed that inhalation of smoke from burning mosquito incense stick or coil containing meperfluthrin leads to liver damage, renal impairment and lung injury in rats [4, 5]. Animal experiments has showed oral lethal dose 50% (LD50) of meperfluthrin is greater than 500 mg/kg, thus oral meperfluthrin mainly causes mild toxic symptoms which can recover well in a short term [1, 4]. Up to now, oral meperfluthrin poisoning in human has rarely been reported. Here, we reported a case of meperfluthrin poisoning by ingestion of a meperfluthrin-based liquid mosquito repellent in a 16-month-old infant. Serious dyspnea and uncontrolled seizures were exhibited.

## Case presentation

The patient was a 16-month-old boy who was admitted to our hospital’s emergency department with a 15-min history of nausea, shortness of breath, and dyspnea. He subsequently developed cyanosis and impairment of consciousness. Foreign body inhalation had initially been diagnosed and the Heimlich maneuver served as the urgent treatment, but no foreign body was coughed up. On physical examination, he was unconscious, bilateral pupil diameter was 3 mm and sluggishly reactive to light. He exhibited rapid and shallow breathing with nasal flaring. The three concave signs were positive. Lung auscultation revealed clear breath sounds present bilaterally. His pulse rate was 161 per minute, and the blood pressure was 75/35 mmHg. Generalized tonic-clonic convulsions were observed after 5 min of admission in the emergency ward. 10% chloral hydrate 5 ml (5 mg/kg) was firstly administered via enema. After intravenous access was available, 2 mg midazolam was administered intravenously twice with intervals of 5 min, as well as intramuscular injection of phenobarbital 0.1 g. Despite of these interventions, the patient kept having uncontrolled convulsions for about 35 min, then 20 mg propofol by intravenous infusion was given to finally control his seizures. The patient’s trachea was intubated during the treatment, and then he was transferred to the intensive care unit for mechanical ventilation. During the hospitalization, meperfluthrin poisoning was considered when his parents provided a critical information that they found an empty liquid mosquito repellent bottle (Jiaojie®) in the location where the patient’s symptoms onset. According to the instruction, nearly 30 ml of meperfluthrin containing 8 mg / ml, total dose of 240 mg, was ingested.

**Fig. 1:**
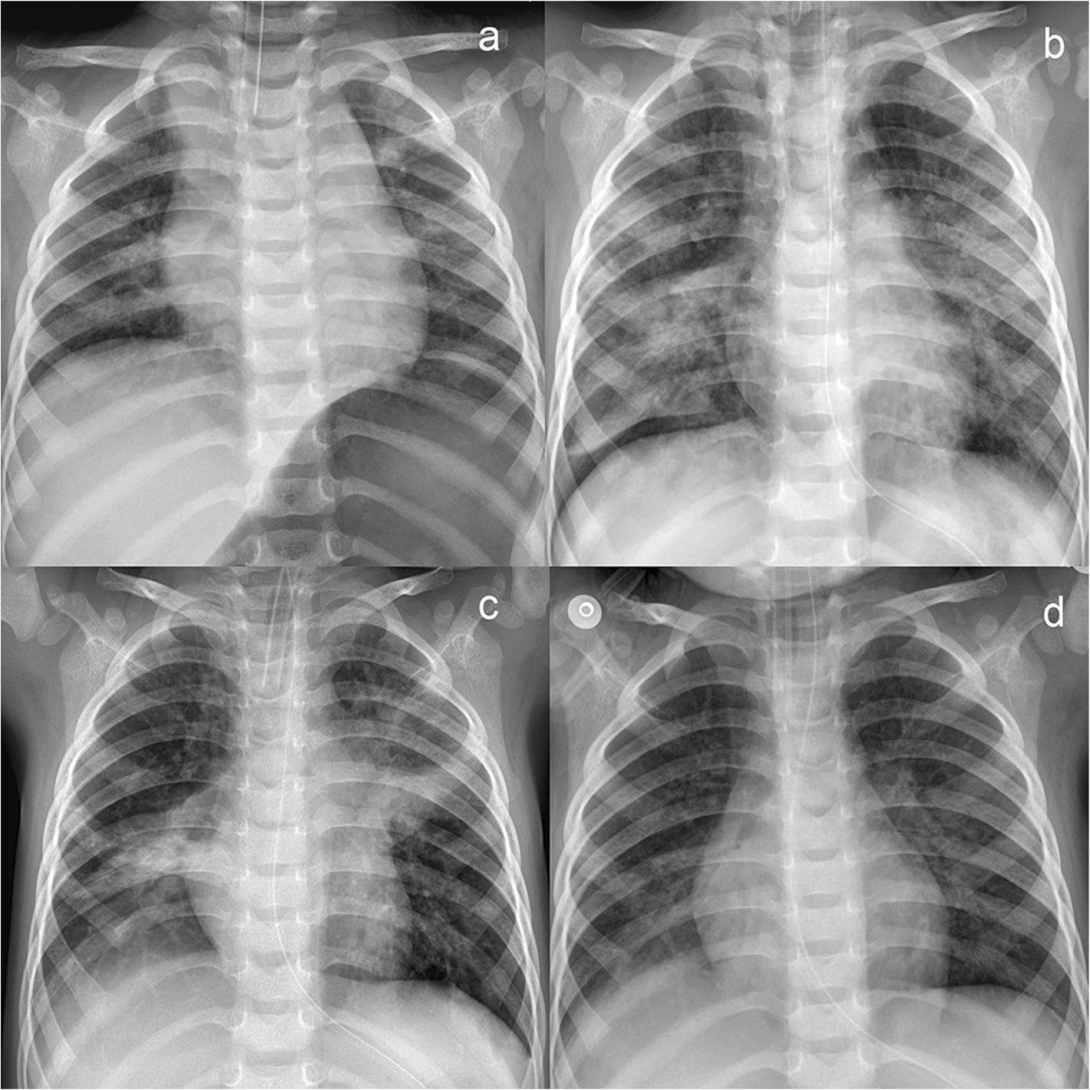
Chest radiograph taken after admission: a. a little bilateral pulmonary exudate showed on day 1; b. multiple patchy and cord-like high-density shadows in the lungs bilaterally on day 2; c. consolidation and atelectasis in the right lower lobe and left upper lobe on day 5; d. a significant improvement of the bilateral pulmonary exudate on day 20

**Fig. 2:**
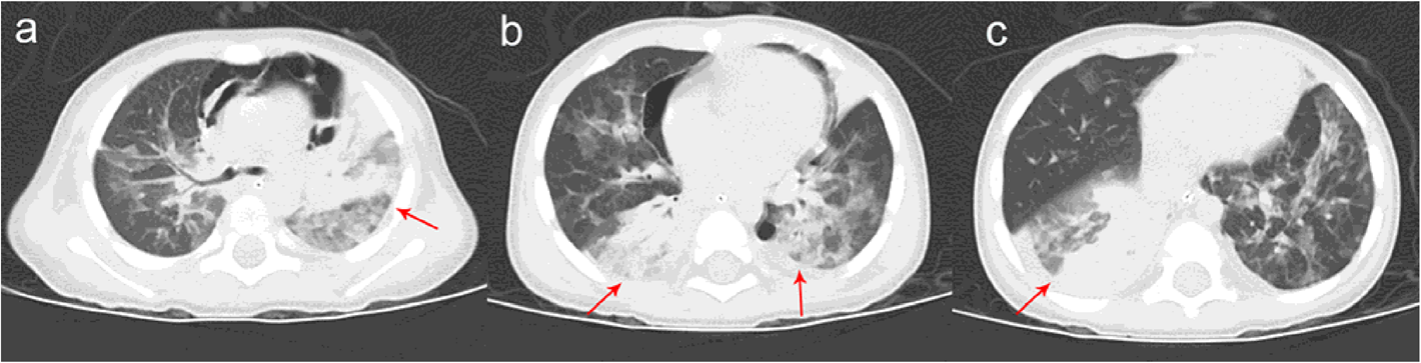
Chest computed tomography taken on day 3 after admission: consolidation and atelectasis were seen in the lower lobe of right lung and upper lobe of left lung as shown by arrow direction, and multiple striated exudation was shown in both lungs

**Fig. 3:**
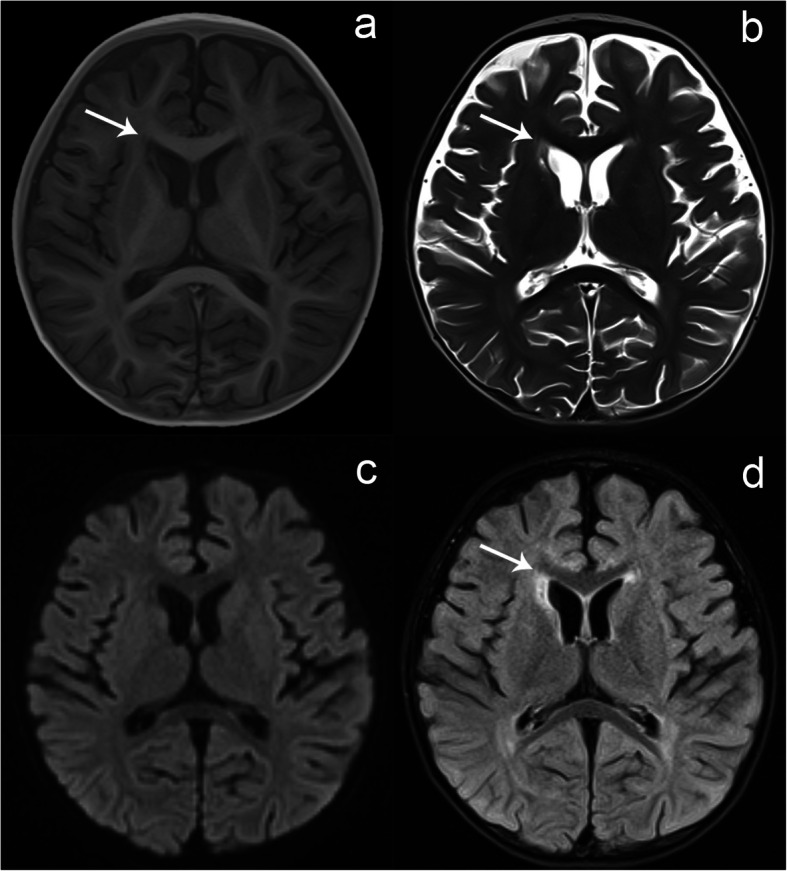
Brain magnetic resonance imaging: a. T1; b. T2; c. DWI; d. T2 Flair. As shown by arrow direction, high signal intensity beside the anterior horn of the right lateral ventricle was seen on the T2-weighted and FLAIR image, low-signal intensity on T1-weighted images

After hospitalization, he presented with fever and had a total leucocyte count of 23,600/mm^3^ and C-reactive protein (CRP) of 116.4 mg/L on day 1. The chest radiograph (as arrowed in Fig. 1a) only revealed a little bilateral pulmonary exudate. Serum electrolytes, lactate dehydrogenase (LDH), creatine phosphokinase (CRK), alanine aminotransferase, aspartate aminotransferase and creatinine level were normal. Cholinesterase was 6188 U/L. Initial arterial blood gas (ABG) analysis showed pH 7.218, pO2 76 mmHg, pCO2 48.1 mmHg, HCO3 19.6 mmol/L, base excess − 8 mmol/L, plasma lactate 4.28 mmol/L. Cerebrospinal fluid (CSF) analysis and cranial computed tomography (CT) showed no abnormalities. On the second day of hospitalization, X-ray chest radiograph (as arrowed in Fig. 1b) showed multiple patchy and cord-like high-density shadows in the lungs bilaterally. Meanwhile, auscultation of the lungs revealed coarse crackles and wheezes. On day 3, Chest CT (Fig. 2) revealed consolidation and atelectasis in the lower lobe of right lung and upper lobe of left lung, and multiple striated exudation could be seen in both lungs. On day 5, his X-ray chest radiograph (as arrowed in Fig. 1c) displayed consolidation and atelectasis in the right lower lobe and left upper lobe. His brain magnetic resonance imaging (MRI) (as arrowed in Fig. 3) showed abnormal signal beside the anterior horn of the right lateral ventricle, and electroencephalogram (EEG) showed the diffused 2.0–4.0 Hz slow wave activity. Fiberoptic bronchoscopy revealed edema and congestion of mucous membrane without erosion or ulcers. The sputum culture gave a positive result for Hemophilus influenzae.

According to the clinical symptoms, laboratory results and imaging findings, the patient was diagnosed with meperfluthrin poisoning, status epilepticus and severe pneumonia. Methylprednisolone (1 mg/kg every 12 h) by intravenous drip infusion and aerosolized beclomethasone dipropionate were prescribed to reduce pulmonary inflammation. Cefoperazone-sulbactam (60 mg/kg every 8 h) was administrated to control lung infection. Mannitol and monosialoteterahexosyl ganglioside were used to reduce intracranial pressure and protect injured nerve cells. He was successfully weaned off from the ventilator on day 15. Routine blood tests and CRP revealed no abnormalities after retesting. After a recheck on day 20, the chest X-ray radiograph (Fig. 1d) showed a significant improvement of the bilateral pulmonary exudate. After obvious symptoms relief, he was transferred to the rehabilitation department and the hyperbaric oxygen therapy was utilized for further neural repair. After a psychiatric consultation, he was discharged from the hospital on day 30. No symptoms had occurred after 12 months of follow up. The chronology of events was showed in Table 1.

**Table 1:** The Chronology of Events

15th min	Nausea, shortness of breath, and dyspnea
20th min	Tonic convulsions
1th h	Transferred to the intensive care unit and treated with mechanical ventilation
24th h	A little bilateral pulmonary exudate on the chest radiograph, no abnormalities on cranial CT and CSF
48th h	Multiple patchy and cord-like high-density shadows on the chest radiograph,
5th day	Consolidation and atelectasis in the right lower lobe and left upper lobe on the chest radiograph, abnormal signal beside the anterior horn of the right lateral ventricle and in parietal paraventricular area on brain MRI, diffused 2.0–4.0 Hz slow wave activity on EEG
20th day	Improvement of the bilateral pulmonary exudate on the chest radiograph
30th day	Discharge

## Discussion and conclusions

In order to answer the clinical question “what are the possible clinical characteristics following meperfluthrin poisoning in chidren?”, MeSH terms “poisoning”, “pyrethrins” and “child” were searched in PubMed. We also used keywords “poisoning”, “meperfluthrin” and “toxicity” to search in Google Scholar. The initial search identified 255 studies. However, there are no reports about meperfluthrin poisoning in children. To our knowledge, our case report is the first one to describe the characteristics of meperfluthrin poisoning in a chid. Although the patient’s total oral dose of meperfluthrin was about 11 mg/kg far below the LD50 in rats, he performed severe respiratory and neurological symptoms. That may attributable to age-related sensitivity to pyrethroids [6]. Unfortunately, although we systematically reviewed clinical data, any blood or urine was not sent for analysis of metabolites of meperfluthrin. The following reasons may explain this. First, due to the lack of a lab for intoxicants identification, clinical specimens from our emergency patients are not routinely sent out for intoxicants identification unless an exposure history to toxicants has been confirmed. Second, the attending physicians might think it was too late for identification of meperfluthrin when his parents provided information about exposure to the intoxicant.

Our reported patient primarily performed severe dyspnea followed by cyanosis which occurred so fast that he was initially diagnosed with foreign body inhalation. However, no foreign body was found when the Heimlich maneuver was given, and the following finding of fiberoptic bronchoscopy did not support inhalation of pyrethroids or other foreign body. Although the patient had a diagnosis of severe pneumonia according to his respiratory symptoms and signs, a positive sputum culture and lung imaging performance, lung infection of Hemophilus influenzae could not fully explain the obvious chest radiographic changes which appeared within 2 days. The patient also had no known history of congenital lung and heart diseases. To sum up, it can be inferred that ingestion of meperfluthrin leads to acute lung injury. Since meperfluthrin synthesis is based on the replacement of the cyano group commonly used in high-efficiency pyrethroids [1], its toxic effects on lungs may be similar to some pyrethroids. It is known that pyrethroids probably disrupt the osmotic gradient generated by both type I and type II pneumocytes, by affecting sodium channels, which leads to mucosal edema in airways at all the levels [7]. As showed in Table 2, George et al. have reported a 30-year old woman with ingestion of 15 mL of prallethrin developing acute respiratory distress syndrome, with X ray and CT of the chest revealing widespread confluent areas of consolidation, and she was in good health after treatment with two weeks of broad spectrum antibiotic and five days of intravenous methylprednisolone [8]. However, oral meperfluthrin poisoning experiments in rats has revealed no abnormalities in the histological sections of lung tissue [4]. We consider the reason may be the inherent differences between human and mouse lungs that lead to different manifestations following acute oral meperfluthrin poisoning.

**Table 2:** Literature regarding uncontrolled seizures and/or pulmonary manifestations induced by ingestion of pyrethroids, by first author

First author, year[Ref.]	Region	Age	Gender	Pyrethroids	Dose(mg)	Symptoms	Chest X-ray/CT	neuroimaging	treatment	prognosis
Jisa George, 2015 [8]	India	30 years	Female	Prallethrin	Unknown quantity	Shortness of breath, chest pain, acute respiratory distress syndrome	X-ray and CT of chest showed widespread confluent areas of consolidation with interlobular septal thickening	Not mentioned	Broad spectrum antibiotics, steroid therapy	Recovery after 21 days
A. Giampreti, 2013 [9]	Italy	19 months	Female	A mixture of bifenthrin and esbiothrin	Unknown quantity	Progressive drowsiness, recurrent tonic–clonic seizures	Not mentioned	No abnormalities	No response to midazolam but thiopental sodium was effective, mechanical ventilation	Recovery after 12 days
Kiran Lata Shringi, 2015 [10]	India	25 years	Female	Transfluthrin	792	Altered sensorium, generalized tonic-clonic convulsions	No abnormalities	No abnormalities	No response to phenytoin and diazepam but a combination of midazolam, propofol and phenobarbitone elicited a response, mechanical ventilation, antibiotics	Recovery after 7 days
Supradip Ghosh, 2009 [11]	India	24 years	Female	Deltamethrin	Unknown quantity	Generalized tonic-clonic seizures	No abnormalities	Not mentioned	No response to laurazepam and phenytoin but a combination of midazolam and thiopentone sodium was effective, mechanical ventilation	Recovery after 5 days
Present study	China	16 months	Male	Meperfluthrin	240	Shortness of breath, serious dyspnea, cyanosis, tonic-clonic convulsions	X-ray and CT of chest showed multiple patchy high-density shadows in the lungs bilaterally.	No abnormalities	No response to 10% chloral hydrate, midazolam and phenobarbital but propofol was effective, broad spectrum antibiotics, mechanical ventilation	Recovery after 30 days

According to recent reports showed in Table 2, oral pyrethroids poisoning can induce severe neurological symptoms, mainly showing uncontrolled seizures which can be controlled only by propofol, thiopentone or combination of benzodiazepines with propofol or thiopentone [9–11]. As is well known, pyrethroids can cause sustained muscle contraction by effects on sodium channels of the spinal cord and peripheral nerves, and pyrethroids also affect the gamma-aminobutyric acid (GABA)-gated chloride channels inducing symptoms such as salivation, myotonia and seizures [12], which may partly explain the molecular mechanisms of meperfluthrin poisoning. Our patient also presented as uncontrolled seizures that the general use of 10% chloral hydrate, midazolam or phenobarbital failed to control. It was noteworthy that the seizures caused by oral meperfluthrin dose far below the LD50 were hardly controlled until propofol was used by intravenous injection. Normal cholinesterase levels excluded exposure to organophosphates. Exposure to certain stimulants such as methamphetamine and methylphenidate could cause seizures, but the present patient had no history of stimulants exposure and his normal CPK and LDH did not support the diagnosis of this type of poisoning [13]. Other differential reasons such as febrile seizures, epilepsy and encephalitis were also excluded because cranial CT scan and CSF analysis were normal and he had no history of pre-existing seizures, neurological diseases, or epilepsy. The abnormal signal beside the anterior horn of the right lateral ventricle on cranial MRI and abnormalities of EEG were considered to be attributed to brain hypoxia due to status epilepticus lasting too long. The initial ABG revealed metabolic acidosis suggesting hypoxia was too long. Therefore, timely and effective oxygen support is critical during his whole treatment period.

The patient we reported showed no symptoms during 12 months of follow-up, which is consistent with the former study that most patients with pyrethroids poisoning have no longstanding or residual symptoms during follow-up [14].

Meperfluthrin poisoning is rare. Acute oral meperfluthrin poisoning can show neurotoxic effects and pulmonary toxicity, and neurotoxic manifestations can be presented as tonic-clonic seizures that the general use of 10% chloral hydrate, midazolam or phenobarbital fail to control. Controlling seizures rapidly and ensuring an adequate oxygen supply are critical to the successful treatment. The underlying mechanisms of meperfluthrin poisoning require further study.

## Data Availability

Data sharing is not applicable to this report as no data sets were generated or analyzed.

## Abbreviations

Co. Ltd.: Company Limited
LD 50: lethal dose 50%
AGB: arterial blood gas
CRP: C-reactive protein
CSF: cerebrospinal fluid
CT: computed tomography
MRI: magnetic resonance imaging
EEG: electroencephalogram
GABA: gamma-aminobutyric acid
LDH: lactate dehydrogenase
CPK: creatine phosphokinase
